# Identification of two mutation sites in spike and envelope proteins mediating optimal cellular infection of porcine epidemic diarrhea virus from different pathways

**DOI:** 10.1186/s13567-017-0449-y

**Published:** 2017-08-30

**Authors:** Min Sun, Jiale Ma, Zeyanqiu Yu, Zihao Pan, Chengping Lu, Huochun Yao

**Affiliations:** College of Veterinary Medicine, Nanjing Agricultural University, Nanjing, Jiangsu China

## Abstract

Entry of the α-coronavirus porcine epidemic diarrhea virus (PEDV) requires specific proteases to activate spike (S) protein for the membrane fusion of the virion to the host cell following receptor binding. Herein, PEDV isolate 85-7 could proliferate and induce cell–cell fusion in a trypsin independent manner on Vero cells, and eight homologous mutation strains were screened by continuous proliferation in the absence of trypsin on Vero cells. According to the whole genome sequence comparative analysis, we identified four major variations located in nonstructural protein 2, S, open reading frame 3, and envelope (E) genes, respectively. Comparative analyses of their genomic variations and proliferation characteristics identified a single mutation within the S2′ cleavage site between C30 and C40 mutants: the substitution of conserved arginine (R) by a glycine (G) (R895G). This change resulted in weaker cell–cell fusion, smaller plaque morphology, higher virus titer and serious microfilament condensation. Further analysis confirmed that this mutation was responsible for optimal cell-adaptation, but not the determinant for trypsin-dependent entry of PEDV. Otherwise, a novel variation (16–20 aa deletion and an L25P mutation) in the transmembrane domain of the E protein affected multiple infection processes, including up-regulation of the production of the ER stress indicator GRP78, improving the expression of pro-inflammatory cytokines IL-6 and IL-8, and promoting apoptosis. The results of this study provide a better understanding of the potential mechanisms of viral functional proteins in PEDV replication, infection, and fitness.

**Electronic supplementary material:**

The online version of this article (doi:10.1186/s13567-017-0449-y) contains supplementary material, which is available to authorized users.

## Introduction

Porcine epidemic diarrhea (PED), characterized by watery diarrhea and dehydration, results in significant economic losses in the swine industry worldwide [1–3]. The causative agent, porcine epidemic diarrhea virus (PEDV), was identified as a member of the alphacoronavirus from the family *Coronaviridae* [1]. The PEDV genome is approximately 28 kb in length, comprising 7 open reading frames (ORF): ORF 1a/1b, spike (S), ORF3, envelope (E), membrane (M) and nucleocapsid (N), in that order [4]. In the process of cell culture and clinical spread of PEDV, several genomic sites show variation and recombination, which are closely related with PEDV cell adaptation, pathogenicity, and evolution [5, 6].

The PEDV spike (S) glycoprotein was recognized as a class I fusion protein, and could be divided structurally into the S1 and S2 regions, mediating the receptor binding and membrane fusion, respectively [7]. Therein S2 contains the S2′ cleavage site, fusion peptide (FP), heptad repeat region 1 (HR1), HR2, and transmembrane domain (TM), in that order [8]. The propagation of PEDV isolates requires supplementation with trypsin in the cell culture supernatant in vitro [1]. Trypsin cleaves the S protein, allowing a conformational change and then mediating fusion activity [8, 9]. The S1/S2 junction and S2′ location have been identified as the important protease cleavage sites [10]. The S2′ cleavage site, FP position, and HR1 domain have been predicted as the determinants for trypsin-dependent entry, and the trypsin-induced cell–cell fusion is weakened by mutation of the S2′ site [8].

The membrane vesicles for coronavirus’ transcription, replication, and generation of new virus particles could be derived from the endoplasmic reticulum (ER) of infected cells [11]. The ER is likely to be overloaded by the extensive use of intracellular membranes, which would cause ER stress responses [11]. Subsequently, the unfolded protein response (UPR) would be triggered to restore the ER homeostasis via three ER-resident transmembrane proteins, otherwise cell apoptosis might be induced [12]. In fact, PEDV has been reported to induce ER stress, UPR, and caspase-independent apoptosis [12, 13].

The envelope (E) protein is a small membrane protein that shares low sequence homology among the coronavirus groups [14]. The E protein, especially the transmembrane domain, is involved in ion-conduction properties, and is highly associated with virus maturation, production, efficient release, and virus-host interactions, which are reflected in the cellular stress, UPR, apoptosis, inflammatory response and pathogenicity [11, 15–17]. Different coronaviruses have varied requirements for the E protein in morphogenesis and virus release. The E protein is absolutely essential for transmissible gastroenteritis virus (TGEV) and middle east respiratory syndrome (MERS-CoV), but not for the mouse hepatitis virus (MHV) and severe acute respiratory syndrome virus (SARS-CoV) [14, 18, 19]. In PEDV, the E protein is located in the ER or nucleus, and has been reported to induce ER stress alone in intestinal epithelial cells (IEC) expressing the PEDV E protein [20]. It remains poorly understood whether or how the E protein participates in PEDV production and infection, and what the critical region is.

In this study, PEDV strain 85-7, which could be cultured on Vero cells in a trypsin independent manner, was isolated successfully. We performed mutation screening by continuous proliferation on Vero cells, and used these strains to evaluate PEDV’s genetic stability. We also identified the major variation regions that were active during cell culture, based on whole genome comparison. Furthermore, we compared the proliferation of these virus characteristics, and identified the functional sites involved in PEDV replication, infection, and fitness. The results were helpful in revealing the PEDV infection mechanism, and demonstrate how PEDV employs specific evolutionary strategies to adapt to the host microenvironment for better survival.

## Materials and methods

### Cell cultures and experimental reagents

Vero cells were cultured in Dulbecco modified Eagle medium (DMEM; Hyclone, USA) with 10% fetal bovine serum (FBS; TCB, USA) and maintained at 37 °C with 5% CO_2_. Cyclosporin A (CsA), Z-VAD-FMK (caspase inhibitor), Dimethyl sulfoxide (DMSO), and Cy3-labeled Goat Anti-Mouse IgG (H+L) were purchased from Beyotime (Shanghai, China). The PEDV specific monoclonal antibody 5E2-C4 was made in our laboratory.

### RNA extraction, reverse transcription (RT)-PCR and quantitative real-time PCR (qPCR)

Viral RNA was extracted from homogenized samples or supernatants of PEDV-infected Vero cells using a Viral RNA Mini kit (Geneaid Biotech), according to the manufacturer’s instructions. PEDV positivity was identified by RT-PCR using specific primers (Additional file 1) and determining its sequence (GENEWIZ, China). The virus cDNA synthesis and PCR amplification were performed according to previously published methods [21].

Total RNA of the PEDV-infected cells, or recombinant plasmid transfected-cells was extracted using the RNAiso Plus reagent (Takara, Japan), according to the manufacturer’s protocols. The concentrations of the extracted RNA were measured using a Thermo Scientific NanoDrop 2000c (Thermo Scientific, USA). The gene-specific primers for qPCR are listed in Additional file 1 [20, 22, 23]. The qPCR was performed with SYBR^®^ Premix Ex Taq™ II (Takara, Japan) and was conducted on an ABI 7300 Real-Time PCR System. The β-Actin gene served as the endogenous control [23]. The relative quantities of mRNA accumulation were evaluated based on the 2^−ΔΔ*Ct*^ method compared with mock-treated results [22]. Each sample was run in triplicate.

### Virus isolation and propagation

PEDV isolation from Vero cells was performed as previously described, with some modifications [1]. Intestinal homogenates or fecal samples were prepared as 30% (wt/vol) suspensions, vortexed, and centrifuged at 3000 × g for 10 min. The supernatant was then filtered through a 0.22-μm-pore-size syringe filter (Millipore, USA). Confluent Vero cells were then washed three times with DMEM, and inoculated with 1 mL of the above supernatant and 4 mL of post-inoculation medium [1] by adding trypsin (Gibco, USA) to a final concentration of 2.5, 10, 20, 30 ng/μL, respectively. The cells were incubated at 37 °C with 5% CO_2_ for 3 days. Viral propagation was confirmed by daily observation of the cytopathic effects (CPE), and using RT-PCR, indirect immunofluorescence assay (IFA), and transmission electron microscopy (TEM). If no positive CPE, RT-PCR or IFA staining results was observed within continuously six blind passages, the result was then considered negative.

### Virus titration and growth characteristics

Confluent Vero cells were washed with DMEM three times, and then infected with the parent 85-7 strain (Passage 5, P5) or the isolated mutants at a multiplicity of infection (MOI) of 0.1 in the presence (10 ng/μL) or absence of trypsin at 37 °C for 1 h, after which the incubation virus was discarded. The infected cells were washed three times with DMEM, cultured at 37 °C by adding the maintenance medium (DMEM supplied with 2% FBS), and the culture supernatants were collected at different time points [12, 24, 36, 48, and 60 h post infection (hpi)]. As to the trypsin-dependency assay, Vero cells were infected with parent 85-7 strain at an MOI of 0.1 within different concentrations trypsin or absence of trypsin, and the culture supernatants were collected at 48 hpi. Finally, titers of the culture supernatants at the indicated times were determined by plaque assay on Vero cells, and quantified as plaque-forming units (pfu) per milliliter (mL).

The viral binding or entry assays were performed as previously described [24], with some modifications. For the viral binding assay, cells were incubated with the same amount of parent 85-7 (P5), C30 or C40 viruses at 4 °C for 1 h in the presence (10 ng/μL) or absence of trypsin. After that the samples were washed three times with DMEM, then the binding efficiency was determined by plaque assay. The entry assay was performed following the viral attachment step. The samples were maintained in DMEM at 37 °C for 1 h in the presence (10 ng/μL) or absence of trypsin, and the cells were washed with cold acidic PBS (pH 3.0) to remove the virus binding to the cell membrane, then the entry efficiency was determined by plaque assay.

### Genome sequencing and genetic stability analysis

The culture supernatants of parent strain 85-7 (P5) and the corresponding variant strains were collected and then viral genomes were extracted. The whole genomes excluding the poly(A) tail, were amplified, sequenced, assembled, and analyzed as previously described [21]. The complete genome sequences of these strains were submitted to the GenBank database, with the accession numbers of KX839246 (parent strain 85-7), KY486713 (strain A40), KX839248 (strain B40), KX839249 (strain C30), KY486714 (strain C40), KX839250 (strain D40), and KX839251 (strain E40).

### Computational analysis and multiple sequence alignments

The transmembrane domain of the PEDV E protein was predicted by TMHMM 2.0 [25]. The schematic overview of the PEDV S2 domain was referred to as the SARS-CoV and then drawn to scale [26]. The nucleotides (S gene or the whole genome of PEDV) or amino acid sequence alignments (S or E protein) were performed using ClustalW with default parameters, using reference sequences of strain DR13 (PEDV, JQ023161.1), cell-adapted DR13 (ca-DR13, JQ023162), strain CV777 (PEDV, AF353511), and strain Tor2 (SARS-CoV, NP828851.1).

### Annexin V and propidium iodide (PI) staining assay

Vero cells were mock- or PEDV-infected at an MOI of 0.1 in the absence of trypsin, and then collected at 36 hpi with 0.25% trypsin. The Annexin V and PI staining assays were measured using an Alexa Fluor 488 Annexin V/Dead Cell Apoptosis kit (Vazyme, China), according to the manufacturer’s protocols. A fluorescence-activated cell sorter (FACS) Accuri C6 flow cytometer (BD Accuri) detected the fluorescent signals of Annexin V and PI using channels FL-1 and FL-2 respectively, and the data was then analyzed by CFlow plus software (BD Accuri).

### Immunofluorescence assay (IFA) staining

IFA staining was performed as follows: first, the mock or PEDV-infected Vero cells were fixed with 4% methanal at 37 °C for 10 min, and then permeabilized with 0.1% Triton X-100 at 37 °C for 5 min. Second, the cells were blocked with 1% bovine serum albumin (BSA) at 37 °C for 1 h, and then stained with mouse anti-PEDV monoclonal antibody 5E2-C4 in 1% BSA (1:200, overnight, 4 °C). Third, the cells were incubated with the Cy3-labeled Goat Anti-Mouse IgG (H+L) (1:500, 37 °C, 1 h, in dark) and then stained with DAPI (37 °C, 10 min, in dark). Finally, the stained-cells were examined by fluorescence microscopy (Zeiss, Germany). All the above mentioned solutions were prepared in PBS, and the cells were washed with PBS containing 0.5% Tween-20 for five times between each step. Furthermore, the Vero cells infected with 85-7 parent, C30, or C40 mutant strains were stained with phalloidin to examine the effects on cytoskeletal organization, as described previously [27].

### Transmission electron microscopy

The mock or PEDV-infected Vero cells were collected gently with a rubber policeman, washed twice with cold PBS (with centrifugation at 2500 × g for 5 min between washing), and then fixed in 2.5% glutaraldehyde overnight at 4 °C. The cell pellet was used to prepare thin sections for observation under TEM, as described previously [28]. The final samples were visualized using a Hitachi-7650 transmission electron microscope (Hitachi Ltd., Japan).

### Virus competition assays

Vero cells were co-infected with PEDV 85-7 C30 and C40 mutant strains at an MOI of 0.1. The initial proportion of C30 and C40 was 1:1 or 4:1, respectively [17]. The co-infected supernatants were then collected at 36 hpi, and serially passaged three times. The relative abundance of the C30 and C40 viruses at passage 3 was determined by comparing the plaque size and sequencing the genetic marker of the S2′ cleavage site. The isolation of viral genomes, gene sequencing, and virus titer determination were performed as described above.

### Western blotting assay

Vero cells were infected with PEDV 85-7 parent, C30, or C40 strains at an MOI of 0.1 for 24 h. The mock Vero cells served as the control. Total proteins were harvested with the RIPA Lysis and Extraction Buffer (ThermoFisher) with protease inhibitor (PMSF, 10%), and prepared for western blot as described previously [24]. The anti-GRP78 antibody (Beyotime, China) was used as the primary antibody, and β-Actin served as the loading control.

### Overexpression of PEDV parent and mutant E proteins

The parent or mutant E genes were amplified from the 85-7 parent or C40 strains by RT-PCR, then cloned into the pIRES2-EGFP expression vector to generate PI-E and PI-mE plasmids, respectively. The PCR primers are listed in Additional file 1, and both the parent and mutant E proteins were C-terminally labeled with HA-tag. The recombinant plasmids were transfected into Vero cells using Lipofectamine 3000 (Invitrogen, USA) according to the manufacturer’s instructions. The overexpression of proteins was examined by dual-staining IFA with anti-HA-tag antibody.

### Statistical analysis

All data were analyzed with the GraphPad Prism 5 software. A *P*-value < 0.05 was considered as significant, and labeled with an asterisk in the figures (*).

## Results

### Virus isolation and characterization

**Figure 1:**
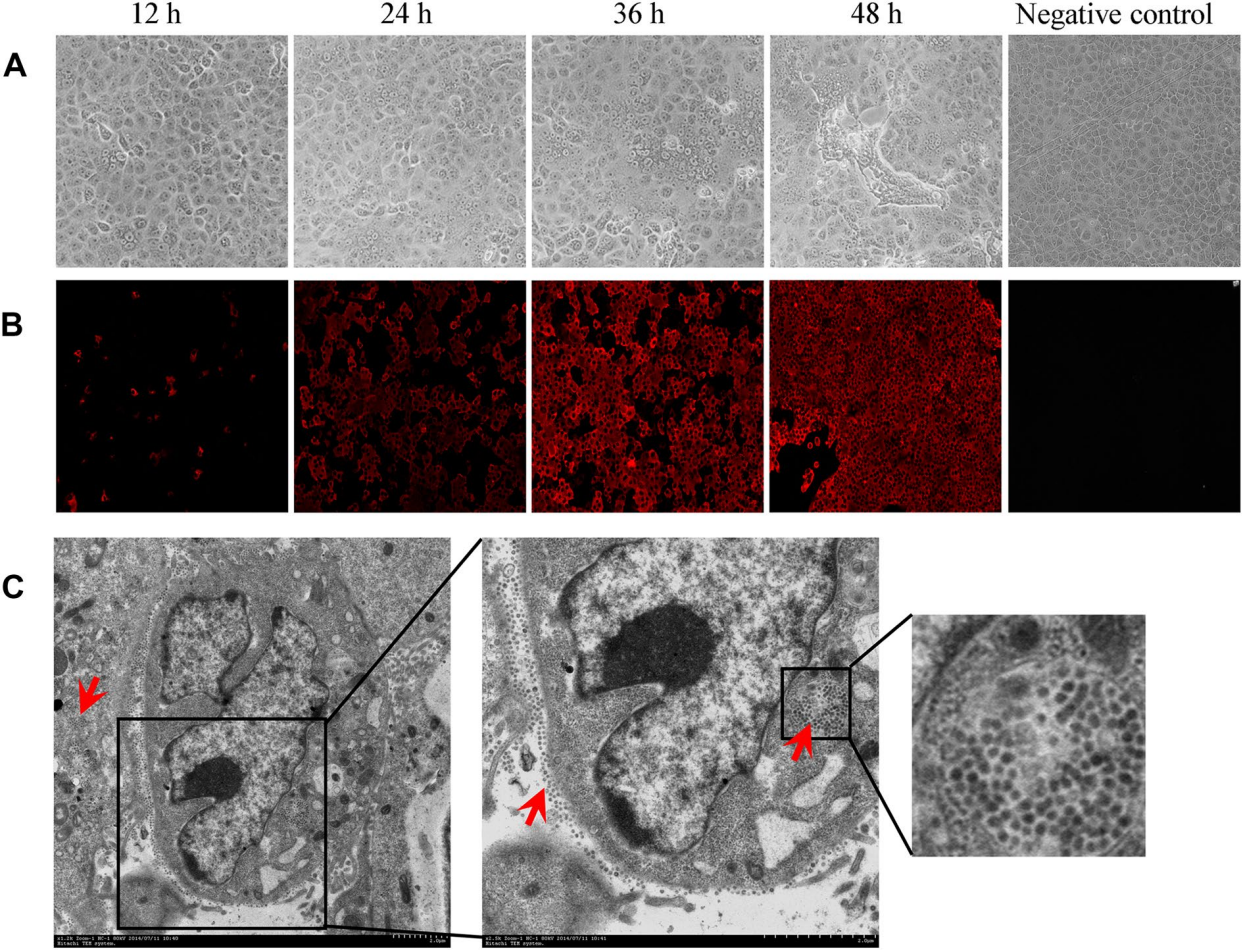
**Infection characteristics of the isolated PEDV strain 85-7.** (**A**) A distinct CPE (P5) was observed and photographed at 12, 24, 36, 48 hpi (magnification of 100×). (**B**) For IFA assay of P5, cells were fixed and incubated with PEDV-specific monoclonal antibody 5E2-C4, followed by Cy3-labeled Goat Anti-Mouse IgG (H+L) (secondary antibody), and then examined by fluorescence microscopy (magnification of 100×). (**C**) A thin section of infected cells (P5) at 24 hpi showing accumulation of virus particles in the cytoplasm membrane and cytoplasm (magnification 1200× and 2500×, Bar = 200 nm. Some virus particles are indicated by red arrows. An enlarged image of part of the cytoplasm is shown.

Fourteen individual PEDV-positive samples were collected and prepared for virus isolation in Vero cells, however, only PEDV strain 85-7 was isolated successfully from the intestinal homogenate. Compared with the negative-control, the Vero cells infected by strain 85-7 (P5) showed a distinct CPE, characterized by cell fusion, and eventually flaking off the culture flask surface (Figure 1A). The CPE was observed at 12 hpi, and became remarkable by 48 hpi (Figure 1A). Each virus passage was assessed using PEDV M gene-based RT-PCR (Additional file 1), and confirmed by gene sequencing (data not shown). Virus propagation was further demonstrated by an IFA assay. The red-stained PEDV were distributed widely in the cytoplasm rather than in the nucleus (Figure 1B). As shown in Figure 1C, numerous virus particles were visible in the infected cells under TEM, which adhered to the cytoplasm membrane and replicated in cytoplasm at 24 hpi.

### Trypsin is not essential for the infection of PEDV strain 85-7 in Vero cells

**Figure 2:**
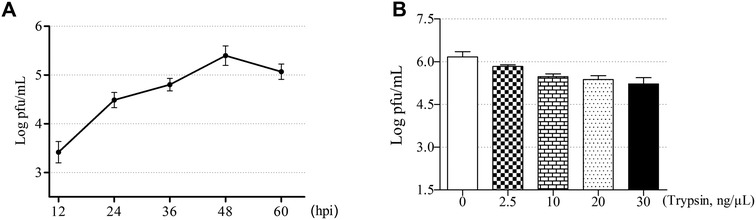
**PEDV strain 85-7 (P5) proliferated on Vero cells in a trypsin independent manner.** (**A**) Virus proliferation curve in the presence of 10 ng/μL trypsin at an MOI of 0.1. Plaque titrations from the indicated time points were carried out. Error bars indicate the standard deviation of the mean of three independent experiments. (**B**) The strain 85-7 could proliferate on Vero cells trypsin-independently. Vero cells were infected at an MOI of 0.1 in the indicated trypsin concentrations (0, 2.5, 10, 20, 30 ng/μL) and the viral titers at 48 hpi were measured and compared.

To further study the proliferation characteristics of strain 85-7, Vero cells were infected with strain 85-7 at an MOI of 0.1 in the presence of trypsin (10 ng/μL). The results show that strain 85-7 proliferated effectively, and reached the peak viral titer (about 10^5.5^ pfu/mL) at 48 hpi (Figure 2A). The essential first step of virus infection is binding to the host cell receptor, and for PEDV, this is related closely to the release of the fusion peptide of the spike protein into target cellular membranes [7]. A large number of host proteases have been identified to activate the CoV-spike protein proteolytically, including the cell surface transmembrane type II serine protease 2 (TMPRSS2), furin, and trypsin [10, 29]. Supplementation with trypsin could assist the virion entry effectively and enhanced their release [8]. By contrast, some PEDV mutants infected Vero cells using a trypsin-independent process [8]. Therefore, the trypsin-dependency of strain 85-7 was further tested, indeed, the virus could infect Vero cells with or without the trypsin in the entry stage (Figure 2B). It should be noted that our results showed a gradual attenuation in the infection of strain 85-7 with increasing trypsin concentration (Figure 2B), as the highest virus titer was reached about 10^6^ pfu/mL in the absence of trypsin at 48 hpi (Figure 2B). These observations indicated that the release of infectious PEDV particles was more efficient without the assistance of trypsin activity, suggesting that strain 85-7 might employ a specific protease cleavage mechanism of the S protein for its infection process.

### Identification of PEDV variants with different infection characteristics by serial passaging in Vero cells

**Figure 3:**
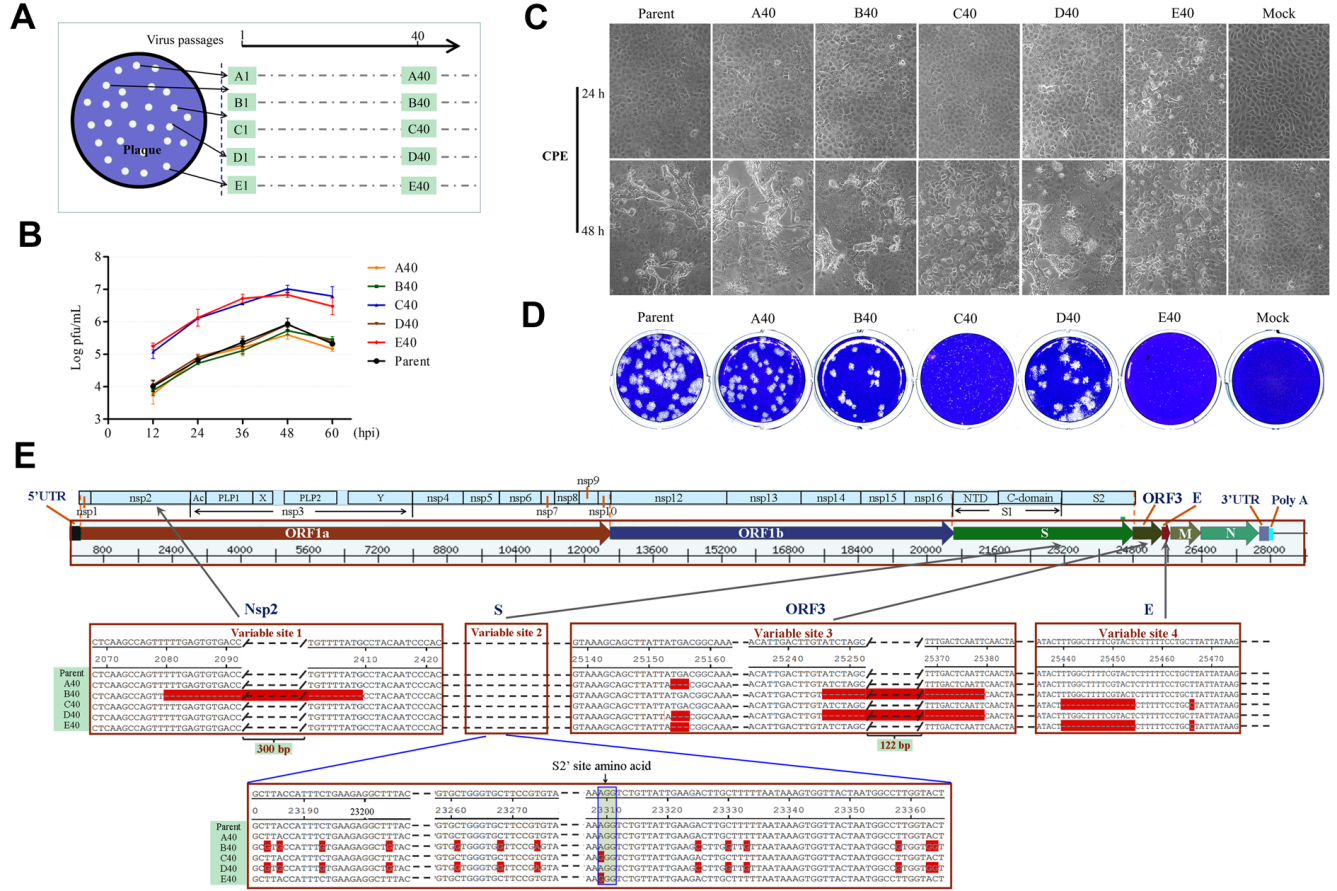
**Identification and comparison of the proliferation characteristics and whole genomes.** (**A**) Serial passage of parent strain 85-7. Five plaques of 85-7 (originated with the same genome) were chosen for serial propagation over 40 passages, which generated five corresponding strains designated as A40, B40, C40, D40 and E40, respectively. (**B**) The virus growth curve of the 85-7 parent strain (P5) and five mutant viruses. Vero cells were infected at an MOI of 0.1 in the absence of trypsin. The supernatant was collected at the indicated times and plaque assays were carried out. (**C**) Comparison of the CPE of 85-7 parent strain (P5) and five mutant viruses at 24 and 48 hpi in the absence of trypsin. The C40 and E 40 strains show weaker cell–cell fusion capacity. (**D**) Comparison of the plaque phenotype of the 85-7 parent strain (P5) and five mutant viruses in the absence of trypsin. The C40 and E 40 strains had distinctly smaller plaques. (**E**) Identification of four major variable regions (V1–V4) in the genomes of the five mutant strains compared with the parent strain.

Previous reports showed that the PEDV genome might display frequent variation in the process of clinical evolution and cell culture, and some of the variant sites are closely related to PEDV pathogenicity and cell adaptation [5, 21], which provides useful clues to explore the molecular functions of viral proteins, and further clarify PEDV’s infection mechanism. Herein, five repeats of strain 85-7 (purified from different virus plaques from the same generation and genetic background) were propagated serially for over 40 passages successfully in Vero cells. For subsequent research, the corresponding strains were designated as A40, B40, C40, D40, and E40 (Figure 3A). Growth kinetic analysis was performed on Vero cells at an MOI of 0.1 under the trypsin-free conditions. Strains A40, B40, and D40 showed a similar growth pattern to the parent virus, while C40 and E40 strains yielded significantly higher titers than other strains during the whole infection process (Figure 3B). The C40 and E40 strains induced CPE morphology of cell pycnosis and non-syncytium formation, which was completely different to that of the other strains (Figure 3C), being consistent with the above observations. Furthermore, the C40 and E40 viruses yielded smaller plaques, even at 5 days post-infection (Figure 3D). All of these observations suggest that C40 and E40 strains might contain some important mutations that contributed to the difference of infection characteristics.

### Mapping the potential genetic determinants for infection characteristics

To evaluate the PEDV genetic stability and explore the potential mechanism that caused the dissimilar infection characteristics in C40 and E40 strains, the whole genomes of the five variant strains and the parent 85-7 were sequenced. The whole genome of the parent 85-7 strain (P5), similar to the published PEDV genomes, was about 27 988 nucleotides (nt) excluding the poly(A) tail, and had a PEDV characteristic gene order of 5′ untranslated region (UTR)-ORF1a/1b-S-ORF3-E-M–N-3′ UTR (Figure 3E).

Comparing the whole genome sequences of the 85-7 parent strain with the five variant strains (A40 to E40), we found that a total of 91 nucleotide sites were changed, leading to 84 amino acid (aa) variations (summarized in Additional files 2 and 3). The analysis of the variable regions of the five mutant strains show that the nucleotide sequences of the non-structural protein Nsp8, Nsp9, and Nsp15 were not subject to any variations during the Vero cell adaptation process of PEDV strain 85-7. Overall, we identified four major variable regions (V1–V4) that contained the most mutation sites (Figure 3E). V1 was located in Nsp2 and comprised a 330 nt deletion (at position 2080–2409) in the C-terminal coding region that led to 110 aa deletion. V2 was located in the S gene and comprised numerous point mutations that were dispersed in the S1 and S2 domains, but with no insertions or deletions. Compared with the parental S gene, there were 7 nt and 27 nt variation points in S1 and S2 regions that induced 6 and 24 aa mutations among the five stable passage strains, respectively. V3 was located in the ORF3 gene with a 3 nt (at position 25 148–25 150) and a 134 nt (at position 25 240–25 373) deletion, causing a 1 aa deletion and the early-termination of encoding mRNA, respectively. V4 was located in the E gene and comprised a 15 nt deletion (at position 25 432–25 446) and 1 nt mutation (T25460C), which caused a 5 aa deletion (16–20 aa) and 1 aa (L25P) mutation in the E protein, respectively (Additional files 2 and 3). The mutation regions of PEDV strain 85-7 varied with unfixed patterns: only two mutation sites were shared among the five variant strains, namely T24530C and G24572C in the S2 region.

**Figure 4:**
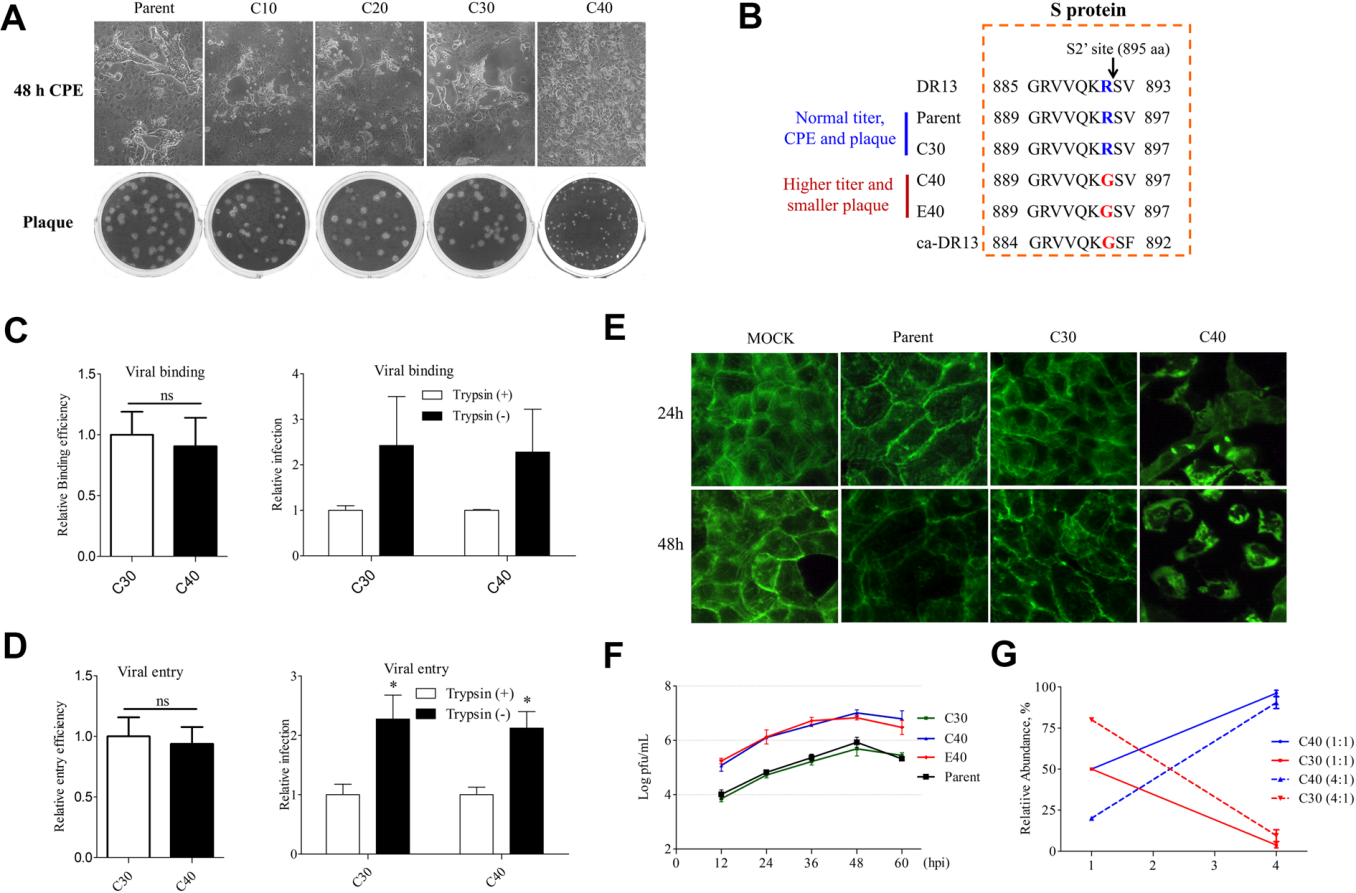
**The effect of mutation R895G of S protein on PEDV replication and viral fitness.** (**A**) Comparison the CPE (48 hpi) and plaque phenotype of 85-7 parent strain (P5) with the precursor strains of C40 strain (strains C10, C20, and C30). (**B**) Alignment of the amino acid sequences of the N-terminal fusion peptide (FP) of DR13, ca-DR13, 85-7 parent, C30, C40, and E40 strains. The ca-DR13, and its parental strain DR13, were reference sequences. The arrow indicated the putative S2′ cleavage site. (**C**) The relative binding efficiency and its trypsin-independence of C30 and C40 strains. (**D**) The relative entry efficiency and its trypsin-independence of C30 and C40 strains. (**E**) Comparison of cytoskeletal reorganization in mock, 85-7 parent, C30, and C40-infected Vero cells. The infected cells were fixed and strained with FITC-Phalloidin at 24 and 48 hpi, respectively, and then photographed under a fluorescent microscope (magnification 400×). (**F**) Comparison of the virus curve of C30 with 85-7 parent, C40, and E40 strains. (**G**) Effect of the PEDV R895G mutation on viral fitness. Competition assays between the C30 mutant virus (red lines) and the C40 virus (blue lines) were performed. Vero cells were co-infected with C30 and C40 viruses at a ratio 1:1 (full line) or 4:1 (dotted line) and supernatants were passaged serially 3 times every 36 h. Error bars represent the standard deviation from three independent experiments.

**Figure 5:**
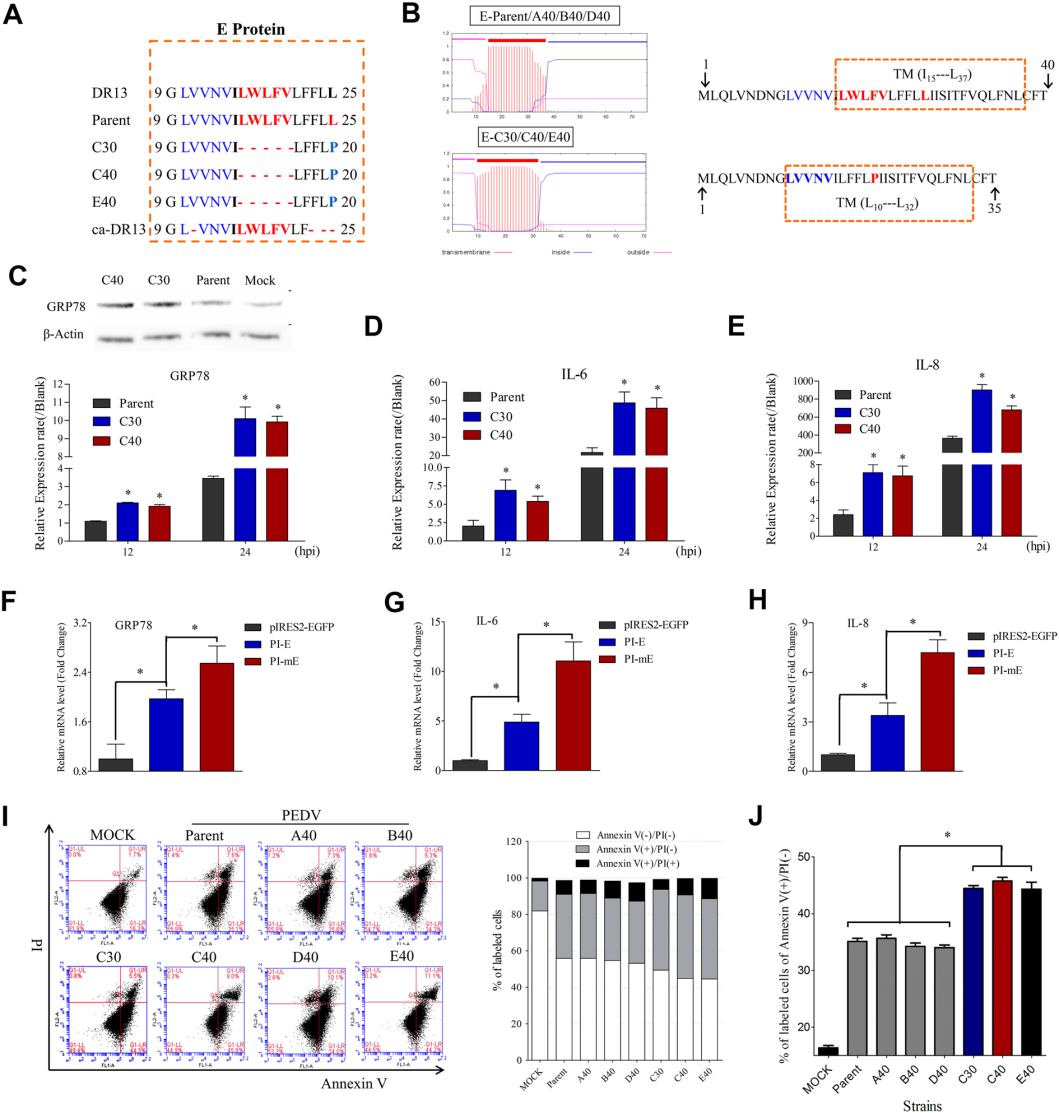
**The variation in the TM domain of E protein played a potential role in PEDV-host interaction.** (**A**) Alignment of the amino acid sequences of the V4 region in E protein from DR13, ca-DR13, 85-7 parent, C30, C40, and E40 strains. C30, C40, and E40 shared the same variation, with a deletion of ^16^LWLFV^20^ and the L25P mutation. (**B**) Analysis of the putative TM domain of the PEDV parent and variant E protein with TMHMM Server v.2.0 [25]. The TM domain was predicted as I_15_ to L_37_ and I_10_ to L_32_, respectively. (**C**) Comparative analysis of the mRNA levels of HSPA5 (encoding GRP78) and the protein production of GRP78 among the parent-, C30-, and C40-infected Vero cells. (**D**, ** E**) Comparative analysis of the mRNA levels of the genes encoding IL-6 and IL-8 among the parent-, C30-, and C40-infected Vero cells by qPCR assay. The β-Actin gene served as an endogenous control. Error bars indicated the means of three independent experiments. (**F** and ** G**) and (**H**) Comparative analysis of the mRNA levels of the genes encoding GRP78, IL-6, and IL-8 among the recombinant plasmids-transfected Vero cells by qPCR assay. The corresponding amount of empty plasmid (pIRES2-EGFP) was used as the mock control. (**I**) Comparison of the cell apoptosis level by flow cytometry with dual Annexin V-PI cell labeling. The infected cells were collected at 36 hpi, and the mock-infected cells were used as the control. Fluorescence-activated cell sorting was used to detect the fluorescent signals of Annexin V and PI, using channels FL-1 and FL-2, respectively. The figure was representative of two independent experiments. The graph representing the percentage of fluorescent signals of Annexin V and PI in each quadrant was shown on the left. (**J**) Comparative analysis of the levels of early apoptosis. C30, C40, and E40 showed higher early apoptosis levels than the 85-7 parent, A40, B40, and D40 strains.

To search for potential sites that contributed to the different infection characteristics, we focused on the identification of specific mutations in the C40 and E40 genomes. The shortened ORF3 with a deletion of 3 nt in A40, D40, and E40 strains; the early-terminated ORF3 with a 134 nt deletion in B40 and D40 strains; and the shortened Nsp2 with a deletion of 330 nt in B40 strains, were judged not to be involved in the infection characteristics of PEDV strain 85-7 (Figures 3, 5I and J). Given that the S protein plays a key role in the coronavirus infection process [26], we identified important sites in the S protein of the five variant strains. The fusion peptide (FP) residues located immediately on the C-terminus of the S2′ cleavage site are very highly conserved across all CoV [30]; however, strains B40 and D40 showed the mutations from ^898^IEDLLF^903^ to ^898^IEALVV^903^ in the FP domain (Additional file 4), which had no significant difference in infection characteristics between them and the parent strain (Figures 3, 5I and J). The only mutational position shared by strains C40 and E40 strains in S protein was the substitution of conserved arginine (R) by a glycine (G) in the S2′ cleavage site (R895G) (Figures 3E and 4B; Additional files 2 and 3). Notably, this site has been reported to be associated with trypsin-induced cell–cell fusion in the PEDV infection process [8], and also serves as the location of the putative cleavage site within the S2 subunit of SARS-CoV and infectious bronchitis virus (IBV) S protein [10, 31]. In addition, the C40 and E40 strains also shared a novel variation region in the E gene, namely V4 region with a 15 nt deletion and a 1 nt mutation (Figure 3E; Additional file 2).

### The R895G mutation of the S2′ site is crucial for virus replication and infection

To identify the decisive sites that mediated the change in infection characteristics of the C40 and E40 strains, we compared the infection characteristics of the precursor strains of C40 strain, namely the 10^th^ (C10), 20^th^ (C20), and 30^th^ (C30) generation strains, with the parent strain. As shown in Figure 4A, the CPE and plaque morphologies of the C10, C20, and C30 strains were the same as the parent strain. Using the close strains C30 and C40 for comparative genomic analysis to avoid the occurrence of chaotic mutations, we managed to identify the sites involved in the above phenotypes. Over the whole genome of the C30 and C40 strains, there was just one mutation (R895G) located in the S2′ site (Figure 4B; Additional files 2 and 3).

In fact, a specific region (named as Part A), including putative S2′ cleavage site, fusion peptide and HR1 domain, has been confirmed as the determinant for PEDV trypsin-dependent entry [8]. The single mutation R895G in putative S2′ cleavage site was speculated to play a key role for this phenotype [8], addressing the difference of biological activities between C30 and C40 strains would define the real function of this novel site. Thus we managed to compare their virus titers on the entry step in the presence or absence of trypsin. The results show that the R895G mutation had no effect on viral binding and entry efficiency (Figures 4C and D), and the presence of trypsin could significantly reduce the infectivity of both C30 and C40 strains in the entry stage (Figures 4C and D), indicating that the S2′ site was not the determinant for trypsin-dependent entry.

We next asked why the R895G mutation caused the more significant cell pycnosis and non-syncytium formation in CPE morphology. To do so we tested the cell microfilament rearrangement after virus infection. As shown in Figure 4E, the control cells were filled with extending microfilaments that formed a complex network, while the parent- and C30-infected cells showed diffuse microfilaments in cytoplasm after 24 hpi, which disappeared significantly at 48 hpi. However, the microfilaments of the C40-infected cells were condensed throughout the cytoplasm and formed bright foci at 48 hpi, indicating that R895G mutation was involved in the microfilaments remodeling of PEDV infected cells.

As to the S2′ site mutant strains C40 and E40 showed higher virus titers than parent and other mutant strains, then we asked whether the S2′ site was involved in PEDV replication. Indeed, the growth curve of C30 was also consistent with the parent stain, and the virus titers were significantly lower than those of the C40 and E40 strains (Figure 4F). Although C40 showed higher virus production and dissimilar infection characteristics in cell culture, it remains unknown whether these differences could improve viral fitness. Thus, we performed a competition assay between the C30 and C40 strains at ratios of 1:1 or 4:1. Through serial subculture three times, the abundance of the C30 strain decreased significantly and was always outcompeted by the C40 strain (Figure 4G), which showed a distinct growth advantage, indicating that strain C40 had evolved for better adaptation to cell culture. All the above results suggest that the R895G mutation of the S2′ cleavage site was the genetic determinant mediating the difference of CPE characteristics, plaque size, growth capability, and cell adaptation between the C30 and C40 strains.

### The novel variation in transmembrane domain of E protein is responsible for stronger ER stress response, inflammatory effect, and higher apoptosis level

It should be noted that the strains C30, C40, and E40 also shared the same variation in E protein with a 5 aa deletion and a 1 aa mutation (Figures 3E and 5A). The PEDV parent E protein contains 76 aa and is predicted to be a transmembrane protein. However, the variant E protein (C30, C40, and E40) was just 71 aa, and its predicted TM domain position was changed to L_10_ to L_32_ (23 aa) (Figure 5B). In fact, the TM domain structure was predicted to be more integral in the variant E protein, while its N-terminal domain was 5 aa shorter compared with the parent E protein (Figure 5B). The coronavirus E protein, especially the TM domain, is involved in many biological processes, including ion channel activity, stress response, and apoptosis [11, 17, 32, 33], thus its potential role in the cell-adaptation process of PEDV could not be overlooked.

A broad range of stresses, including virus infection, can perturb ER function seriously [12]. The intracellular stress response UPR was triggered to recover ER homeostasis and initiate inflammation through the release of inflammatory cytokines [12, 34]. GRP78 is a well-characterized ER chaperone protein and a marker of ER stress [12]. Our results show that C30 and C40 strains significantly activated *HSPA5* (the gene encoding GRP78) transcription and GRP78 production during the virus replication process compared with those of the parent strain (Figure 5C). Meanwhile, the transcriptions of pro-inflammatory cytokines IL-6 and IL-8 were also significantly up-regulated in C30 and C40 infected cells than parent virus infected cells (Figures 5D and E). Further study showed that overexpression of mutant E protein could induce significantly higher mRNA level of IL-6, IL-8, and GRP78 than parent E protein (Figures 5F–H; Additional file 6). These results indicate that the variation in the TM domain of the E protein caused a notable effect on its interaction with the host cells.

It should be noted that the continuous or too strong stimulation of ER stress can induce cell apoptosis [11]. Thus, we tested the apoptosis levels of the parent and mutant strains using flow cytometry. Compared with the mock-infected cells, the parent 85-7 strain triggered a positive result for caspase-independent apoptosis, with the percentage of early apoptotic cells (Annexin V positive/PI negative) increasing from 16.3 to 35.1% (Figure 5I; Additional file 5). Notably, the C30, C40, and E40 strains induced significantly higher apoptosis levels than the parent strain (Figures 5I and J). Furthermore, the similar apoptosis levels induced by the C30 and C40 strains indicated that the R895G mutation might not contribute to this pathogenic pathway (Figures 5I and J). The apoptosis levels induced by strains A40, B40, and D40 strains also showed no difference compared with the parent strain (Figures 5I and J), further confirming that the other mutations were not involved in this phenotype.

## Discussion

In recent years, PEDV has reemerged in Asia and Europe, and has spread into America and Australia, demonstrating a complex virulence situation, genetic recombination, and evolution [3, 4, 35, 36]. In this study, the infection of PEDV strain 85-7 could significantly up-regulate GRP78 production and the level of the mRNA encoding pro-inflammatory factors, and induced cell apoptosis and microfilament rearrangement on Vero cells, which provided clues to further explore PEDV’s pathogenic mechanism. Furthermore, PEDV undergoes frequent variation in the process of clinical evolution and cell culture, which contributes to its fast adaptation to the host microenvironment [5, 21, 37, 38]. Indeed, genomic stability analysis of PEDV strain 85-7 identified four major variable regions, located in the Nsp2, S, ORF3, and E genes, respectively. Full understanding of the effects of these variations on infection characteristics was necessary to reveal the PEDV infection mechanism and evolutionary trend.

Truncation of the C-terminus of Nsp2 had no distinct effects on PEDV replication (Figure 3). Similarly, mutations in Nsp2 did not affect viral replication but caused a modest reduction in titers and viral RNA synthesis in MHV and SARS-CoV [39]. The protein domains of Nsp1 to Nsp3 homologs showed minimal sequence identity among different coronavirus groups [39]. In fact, Nsp2 is one of the most variable regions, with diverse recombination events being identified in field strains of PEDV [4], but is not involved in the PEDV-induced IFN antagonistic effect [37], all of which suggest that Nsp2 might mediate virus evolution, rather than playing a role in viral replication. Similarly, the early termination of ORF3 gene studied here had no significant effects on PEDV replication, while the function of ORF3 in PEDV replication is controversial and requires further exploration [5, 37, 40–42].

More importantly, the C30 and C40 mutant strains, differing just with R895G mutation, showed significant difference in viral yields, cytoskeletal structure, CPE effects and plaque morphologies. A previous study has reported that the disordered microfilaments induced by PEDV infection in IPEC-J2 cells could suppress the replication and release of viruses [27], which implied that R895G mutation might contribute to higher viral titers via mediation of the cytoskeleton rearrangement. Furthermore, virus infection could also induce cellular conformational changes and cytopathic effect via rearranging host cell cytoskeletal and membrane compartments [43], suggesting that the R895G mutation might be the decisive site that allows the virus to evolve toward better survival in host cells.

Several protease cleavage sites in the S proteins of *coronavirus* have been identified, and most of them play key roles in the virus infection process [8, 10]. The R895 site of PEDV S protein forms the novel XXXR/S motif, and has been identified as a putative S2′ cleavage site, which was predicted as the determinant in trypsin-dependent entry of PEDV [8]. However, PEDV 85-7 parent and all the mutant strains (including the C30 and C40 strains differing single mutation R895G) could effectively proliferate in Vero cells in a trypsin-independent manner, and the entry or release of infectious PEDV particles was more efficiently without trypsin supplementation, indicating that the S2′ site was not the determinant for trypsin-dependent entry of PEDV. Coupled with the reports that R895G mutation in S protein could inhibit cell–cell fusion specifically in both PEDV and SARS-CoV [8, 10], suggesting that the PEDV induced cell–cell fusion capacity might be more closely related with the S2′ cleavage site than with trypsin supplementation. Thus, supplementation with trypsin is required for the virus-cell fusion process of trypsin-dependent PEDV infection, while the cell–cell fusion process might be mediated by the S2′ cleavage site via a completely different mechanism.

The specific region of S protein, including the putative S2′ cleavage site, fusion peptide, and HR1 domain, has been confirmed as the determinant for trypsin-dependent entry of PEDV in the attenuated DR13 strain [8]. An amino acid sequence alignment of this region between trypsin-dependent strains (CV777 and DR13) and trypsin-independent strains (ca-DR13 and 85-7), revealed only one position difference, from Y977 (CV777 and DR13) to H977 (ca-DR13 and 85-7) (Additional file 7). These observations suggest that the Y977H mutation might be the determinant for PEDV trypsin-independent entry and served as a crucial proteolytic cleavage site.

Furthermore, the fusion peptides located close to the C-terminus of the S2′ cleavage site are highly conserved across the *Coronaviridae*, particularly the ^898^IEDLLF^903^ motif, which contains four hydrophobic residues (I, Isoleucine; L, Leucine; F, Phenylalanine) and two negative-charged residues (E, Glutamic acid; D, Aspartic acid) [30]. Notably, the residues ^901^LLF^903^, but not the D^900^, have been shown to have a crucial role in membrane fusion in SARS-CoV [30]. However, the B40 and D40 strains with point mutations comprising D900A (Alanine), L902 V (Valine), and F903 V showed similar infection characteristics to the parent strain (Figure 3; Additional file 4). Alanine (A) and Valine (V) are also hydrophobic residues, which suggests that the occasional conservative substitution in the ^898^IEDLLF^903^ motif caused only a minimal change in PEDV infection.

It is noteworthy that C30, C40, and E40 mutant strains sharing the same variation in TM domain of the E protein could up-regulate the transcription of GRP78, IL-6 and IL-8, and induce higher apoptosis levels compared with that of the parent strain. The SARS-CoV E protein alone was sufficient to reduce the expression of GRP78 and GRP94 (two ER stress inducible proteins) [11], whereas the PEDV E protein could be located in the ER, and then induced ER stress and up-regulated GRP78 alone [20], suggesting that the E proteins from diverse *Coronavirus* viruses might employ different mechanisms to mediate the stress response. Furthermore, SARS-CoV lacking the E gene (rSARS-CoV-ΔE) has been demonstrated to reduce the expression of pro-inflammatory cytokines [11]. In PEDV, the E protein also increased IL-8 expression in intestinal epithelial cells (IEC) [33] and Vero cells (Figures 5E and H), suggesting that the E protein is closely related to inflammation. These studies, coupled with the reports that TM domain alterations of CoV E protein are involved in virus assembly, replication and release [15, 32], supported our speculation that the TM domain of PEDV E protein plays a potential role in interaction with host cells, including ER stress, pro-inflammatory cytokine production and apoptosis. Especially, it is the variation of the E protein, not the amount of virus production, that is responsible for the increase of the cell stress response. Some recent studies have reported that the ion channel (IC) activity of CoV E protein has important functions in immune response modulation via NLRP3 inflammasome, and the IC activity relies on its TM domain [17, 44]. However, it needs further study whether the variation of the TM domain in this study has an impact on the ion channel activity of PEDV E protein and the following immune response modulation.

In conclusion, we performed a comprehensive analysis of the viral proliferation characteristics and the alignment of whole genomes among the isolated PEDV strain 85-7 and several of its mutants screened from serial propagation on Vero cells. The results allowed us to hypothesize that the R895G mutation in S protein is the determinant of PEDV-induced cell–cell fusion, and also promotes PEDV replication and fitness. Furthermore, the E protein, especially the V4 region in the TM domain, is crucial for modulating the PEDV cell stress response and immune response. In short, continuous passage of PEDV strain 85-7 induces the above two variations to adapt the virus to the host microenvironment for better proliferation.

## Additional files

**Additional file 1.** **Primers designed for RT-PCR, qPCR and recombinant vector constructing.**

**Additional file 2.** **Summary of nucleotide changes of PEDV strain 85-7 during serial passages in cell culture.**

**Additional file 3.** **Summary of amino acid variations of PEDV strain 85-7 during serial passages in cell culture.**

**Additional file 4.** **The alignment of the amino acid sequences** ^**898**^ **IEDLLF** ^**903**^ **in the fusion peptide (FP).** Minimal divergence with occasional conservative substitutions in B40 and D40 strains were shown. The amino acids of I, Isoleucine; L, Leucine; F, Phenylalanine; V, Valine were hydrophobic amino acids. The E, Glutamic acid, and D, Aspartic acid were negative-charged amino acids.

**Additional file 5.** **PEDV strain 85-7 induced caspase-independent apoptosis on Vero cells.** (A) IFA of 85-7 parent strain in infected Vero cells with treatment of CsA (10 μM), V-ZAD-FMK (100 μM) and DMSO at 36 hpi. CsA treatment suppressed PEDV replication, while the V-ZAD-FMK had no significant effect on virus growth. Vero cells were pretreated with CsA, V-ZAD-FMK or DMSO for 1 h, and then infected with PEDV with the presence of CsA, V-ZAD-FMK or DMSO in the whole infected process. (B) Viral titers of the infected cells with CsA (1, 5, 10 μM) or DMSO treatment at 12, 24 and 36 hpi. Viral titers were determined as Log pfu/mL. Error bars indicate the average results of two independent experiments. (C) Viral titers of the infected cells with V-ZAD-FMK (100 μM) or DMSO treatment at 36 hpi.

**Additional file 6.** **The dual-staining IFA assay to verify the overexpression of PEDV parent E protein and mutant E proteins in Vero cells.** The recombinant plasmids (PI-E or PI-mE) were transfected into Vero cells for 24 h. The corresponding amount of empty plasmid (pIRES2-EGFP) was used as the mock control. The primary antibody was the anti-HA-tag antibody. The red staining (recombinant protein) was almost merged with the green staining (EGFP-tag protein).

**Additional file 7.** **Multiple amino acid sequence alignment of the determinant region for PEDV trypsin-dependent entry.** A schematic overview of the S2 domain is referred to as the SARS-CoV. FP, fusion peptide; HR1 and HR2, heptad repeat regions; S2′ site (R895), location of putative cleavage site within the S2 subunit; drawn to scale. Virus abbreviations (and GenBank accession numbers) were as follows: SARS-CoV (Tor2, NP_828851.1), DR13 (JQ023161.1), ca-DR13 (JQ023162.1), CV777 (AF353511.1). The Y977H was the only site that differed the trypsin-independent strains (ca-DR13 and 85-7 strain) from the trypsin-dependent strains (DR13 and CV777).

## Abbreviations

PEDV: porcine epidemic diarrhea virus
Nsp: nonstructural protein
ORF: open reading frame
PED: porcine epidemic diarrhea
FP: fusion peptide
HR: heptad repeat region
TM: transmembrane domain
ER: endoplasmic reticulum
UPR: unfolded protein response
TGEV: transmissible gastroenteritis virus
MERS-CoV: Middle East Respiratory Syndrome-coronavirus
MHV: mouse hepatitis virus
IBV: infectious bronchitis virus
SARS-CoV: severe acute respiratory syndrome-coronavirus
IEC: intestinal epithelial cells
DMEM: Dulbecco modified eagle medium
FBS: fetal bovine serum
CsA: cyclosporin A
DMSO: dimethyl sulfoxide
RT-PCR: reverse transcription-polymerase chain reaction
qPCR: quantitative real-time polymerase chain reaction
CPE: cytopathic effects
IFA: indirect immunofluorescence assay
MOI: multiplicity of infection
hpi: hours postinfection
pfu: plaque-forming units
PI: propidium iodide
FACS: fluorescence-activated cell sorter
BSA: bovine serum albumin
PBS: phosphate-buffered saline
TEM: transmission electron microscopy
TMPRSS2: transmembrane type II serine protease 2
UTR: untranslated region
