# Supplementary material for: Identification of two mutation sites in spike and envelope proteins mediating optimal cellular infection of porcine epidemic diarrhea virus from different pathways
Source: Vet Res. 2017 Aug 30;48:44. doi: 10.1186/s13567-017-0449-y (PMC5577753; doi:10.1186/s13567-017-0449-y)
Supplement: Supplementary file 1 — Additional file 1. Primers designed for RT-PCR, qPCR and recombinant vector constructing. [file 13567_2017_449_MOESM1_ESM.docx]

Additional file 1. Primers designed for RT-PCR, qPCR and recombinant vector constructing.

| Primer Name | Sequence (5’-3’) | Reference |
| --- | --- | --- |
| Primer for PEDV-positive identification | |  |
| M-F | CATTCGGTTGTGGCGCAGGAC | This study |
| M-R | CGCCGTGTTTGGACCGGACATA |  |
| Primers for qPCR assay | |  |
| IL-6-F | GCTGCAGGCGCAGAACCA | [[1](#_ENREF_1)] |
| IL-6-R | AAAGCTGCGCAGGATGAGA |  |
| IL-8-F | CTGGCGGTGGCTCTCTTG | [[1](#_ENREF_1)] |
| IL-8-R | CCTTGGCAAAACTGCACCTT |  |
| GRP78-F | AACGGCCGCGTGGAGATCA | [[2](#_ENREF_2)] |
| GRP78-R | GAGCTGGTTCTTGGCGGCAT |  |
| β-Actin-F | CGGGAAATCGTGCGTGAC | [[3](#_ENREF_3)] |
| β-Actin-R | ATGCCCAGGAAGGAAGGTTG |  |
| Primers for recombinant vector constructing | |  |
| E-F | ACGCGTCGACATGCTACAATTAGTGAATGATAA |  |
| E-R | CGCGGATCCTTAAGCGTAGTCTGGGACGTCGTATGGGTA  TACGTGAATAACAGTACTGGG | This study |

1. Jing H, Fang L, Wang D, Ding Z, Luo R, et al. (2014) Porcine reproductive and respiratory syndrome virus infection activates NOD2-RIP2 signal pathway in MARC-145 cells. Virology 458-459: 162-171.

2. Xu X, Zhang H, Zhang Q, Dong J, Liang Y, et al. (2013) Porcine epidemic diarrhea virus E protein causes endoplasmic reticulum stress and up-regulates interleukin-8 expression. Virol J 10: 26.

3. Mu Y, Li L, Zhang B, Huang B, Gao J, et al. (2015) Glycoprotein 5 of porcine reproductive and respiratory syndrome virus strain SD16 inhibits viral replication and causes G2/M cell cycle arrest, but does not induce cellular apoptosis in Marc-145 cells. Virology 484: 136-145.
