# Supplementary material for: Identification of two mutation sites in spike and envelope proteins mediating optimal cellular infection of porcine epidemic diarrhea virus from different pathways
Source: Vet Res. 2017 Aug 30;48:44. doi: 10.1186/s13567-017-0449-y (PMC5577753; doi:10.1186/s13567-017-0449-y)
Supplement: Supplementary file 2 — Additional file 2. Summary of nucleotide changes of PEDV strain 85-7 during serial passages in cell culture. [file 13567_2017_449_MOESM2_ESM.docx]

**Additional file 2.** **Summary of nucleotide changes of PEDV strain 85-7 during serial passages in cell culture.**

| **Position** | | **Parent** | **A40** | **B40** | **C40** | **D40** | **E40** | **C30** |
| --- | --- | --- | --- | --- | --- | --- | --- | --- |
| **nt** | **Gene** |  |  |  |  |  |  |  |
| 100 | 5' UTR | G | G | G | G | G | A | G |
| 301 | Nsp1 | A | A | A | A | A | T | A |
| 1921 | Nsp2 | A | A | G | A | A | A | A |
| 2080-2409 | Nsp2 | / | / | △330nt | / | / | / | / |
| 3469-3470 | Nsp3 | AC | AC | AAAGATAC | AC | AAAGATAC | AC | AC |
| 5957 | Nsp3 | A | A | A | A | C | A | A |
| 9256 | Nsp4 | A | A | G | A | A | A | A |
| 9516 | Nsp5 | A | A | T | A | A | A | A |
| 9732 | Nsp5 | T | T | C | T | T | T | T |
| 11003-11005 | Nsp6 | TTT | GTC | TTT | TTT | ATT | TTT | TTT |
| 11014 | Nsp6 | C | C | C | C | A | C | C |
| 11056-11057 | Nsp7 | TT | TT | TT | TT | CC | TT | TT |
| 11067-11068 | Nsp7 | TT | TT | TT | TT | CA | TT | TT |
| 11110 | Nsp7 | T | T | T | T | C | T | T |
| 11189-11191 | Nsp7 | TTT | TTT | TTT | TTT | CAA | TTT | TTT |
| 12268-12269 | Nsp10 | AA | TT | AA | AA | AA | AA | AA |
| 12552 | Nsp10 | T | C | C | T | C | T | T |
| 14569 | Nsp12 | C | C | T | C | C | C | C |
| 14774 | Nsp12 | C | G | C | C | C | C | C |
| 14811 | Nsp12 | C | G | C | C | C | C | C |
| 14852 | Nsp12 | T | G | T | T | T | T | T |
| 14953 | Nsp12 | C | T | C | C | C | C | C |
| 14977 | Nsp12 | T | G | T | T | T | T | T |
| 15004 | Nsp12 | C | G | C | C | C | C | C |
| 15013 | Nsp12 | C | T | C | C | C | C | C |
| 15029 | Nsp12 | T | T | T | T | G | T | T |
| 15040 | Nsp12 | C | G | C | C | C | C | C |
| 15059 | Nsp12 | T | C | T | T | T | T | T |
| 15652 | Nsp13 | C | T | C | C | C | C | C |
| 17913 | Nsp14 | A | A | A | A | C | A | A |
| 20139 | Nsp16 | G | A | G | A | G | A | A |
| 21604 | S1 | C | C | C | C | C | G | C |
| 21656 | S1 | C | C | C | C | T | C | C |
| 22092 | S1 | A | A | G | A | G | A | A |
| 22263 | S1 | A | A | G | A | G | A | A |
| 22427 | S1 | A | A | G | A | G | A | A |
| 22653 | S1 | C | C | T | C | T | C | C |
| 22836 | S1 | C | C | T | C | C | C | C |
| 23178-23180 | S2 | TTA | TTA | GTG | TTA | GTG | TTA | TTA |
| 23187 | S2 | C | C | G | C | G | C | C |
| 23198 | S2 | T | T | G | T | G | T | T |
| 23255 | S2 | C | C | G | C | G | C | C |
| 23262 | S2 | C | C | G | C | G | C | C |
| 23268 | S2 | T | T | A | T | A | T | T |
| 23303 | S2 | A | A | A | G | A | G | A |
| 23319 | S2 | A | A | C | A | C | A | A |
| 23324 | S2 | C | C | G | C | G | C | C |
| 23327 | S2 | T | T | G | T | G | T | T |
| 23352 | S2 | T | T | G | T | G | T | T |
| 23357-23358 | S2 | AC | AC | GG | AC | GG | AC | AC |
| 23526 | S2 | C | C | C | C | C | G | C |
| 23528 | S2 | G | G | A | G | G | G | G |
| 24172 | S2 | T | T | C | T | T | T | T |
| 24194 | S2 | A | A | A | A | A | G | A |
| 24420 | S2 | A | A | G | A | G | G | A |
| 24447 | S2 | A | A | C | A | C | C | A |
| 24462 | S2 | A | A | C | A | C | C | A |
| 24500 | S2 | T | T | C | T | C | C | T |
| 24510-24512 | S2 | ATT | ATT | CTA | ATT | CTA | CTA | ATT |
| 24523 | S2 | T | T | C | T | C | C | T |
| 24530 | S2 | T | C | C | C | C | C | C |
| 24572 | S2 | G | C | C | C | C | C | C |
| 25148-25150 | ORF3 | TGA | △3nt | TGA | TGA | △3nt | △3nt | TGA |
| 25161 | ORF3 | A | G | A | A | A | A | A |
| 25240-25373 | ORF3 | / | / | △134nt | / | △134nt | / | / |
| 25434-25448 | E | / | / | / | △15nt | / | △15nt | △15nt |
| 25460 | E | T | T | T | C | T | C | C |
| 25653 | M | A | A | A | A | C | A | A |
| 25692 | M | A | A | C | A | A | A | A |
| 25852-25853 | M | GG | AT | GG | GG | GG | GG | GG |
| 26001 | M | C | T | T | C | T | C | C |
| 26380 | N | C | C | C | C | T | C | C |
| 26399 | N | T | G | T | T | T | T | T |
| 26873 | N | A | A | G | A | A | A | A |
| 26895 | N | C | C | T | C | C | C | C |
| 26907 | N | T | T | T | T | C | T | T |
| 27096 | N | C | C | C | C | T | C | C |
| 27536 | N | G | A | G | G | G | G | G |
| 27650 | N | A | G | A | A | A | A | A |
| 27987-27989 | 3' UTR | CCA | ACC | ACC | CCA | ACC | ACC | CCA |

/: the same with the parent strain;

△: nucleotide deletion
