# Supplementary material for: Identification of two mutation sites in spike and envelope proteins mediating optimal cellular infection of porcine epidemic diarrhea virus from different pathways
Source: Vet Res. 2017 Aug 30;48:44. doi: 10.1186/s13567-017-0449-y (PMC5577753; doi:10.1186/s13567-017-0449-y)
Supplement: Supplementary file 3 — Additional file 3. Summary of amino acid variations of PEDV strain 85-7 during serial passages in cell culture. [file 13567_2017_449_MOESM3_ESM.docx]

**Additional file 3. Summary of amino acid variations of PEDV strain 85-7 during serial passages in cell culture.**

| **Position** | | **Parent** | **A40** | **B40** | **C40** | **D40** | **E40** | **C30** |
| --- | --- | --- | --- | --- | --- | --- | --- | --- |
| **Protein** | **Position (aa)** |  |  |  |  |  |  |  |
| ORF1a | 4 | N | N | N | N | N | Y | N |
|  | 544 | I | I | V | I | I | I | I |
|  | 597-706 | / | / | △110aa | / | / | / | / |
|  | 1059-1060 | IT | IT | IKDT | IT | IKDT | IT | IT |
|  | 1889 | D | D | D | D | A | D | D |
|  | 2989 | N | N | D | N | N | N | N |
|  | 3571-3572 | IS | SP | IS | IS | NS | IS | IS |
|  | 3575 | Q | Q | Q | Q | K | Q | Q |
|  | 3589 | L | L | L | L | P | L | L |
|  | 3593 | S | S | S | S | T | S | S |
|  | 3607 | C | C | C | C | R | C | C |
|  | 3633-3634 | FF | FF | FF | FF | SI | FF | FF |
|  | 3993 | K | L | K | K | K | K | K |
| ORF1b | 661 | S | S | F | S | S | S | S |
|  | 742 | R | G | R | R | R | R | R |
|  | 789 | S | F | S | S | S | S | S |
|  | 797 | I | S | I | I | I | I | I |
|  | 806 | S | W | S | S | S | S | S |
|  | 809 | T | I | T | T | T | T | T |
|  | 814 | D | D | D | D | E | D | D |
|  | 818 | T | S | T | T | T | T | T |
|  | 1022 | T | I | T | T | T | T | T |
|  | 1776 | N | N | N | N | H | N | N |
|  | 2518 | E | K | E | K | E | K | K |
| S | 346 | L | L | L | L | F | L | L |
|  | 491 | K | K | R | K | R | K | K |
|  | 548 | K | K | R | K | R | K | K |
|  | 603 | N | N | D | N | D | N | N |
|  | 678 | T | T | I | T | I | T | T |
|  | 739 | S | S | F | S | S | S | S |
|  | 853-856 | LTIS | LTIS | RAIC | LTIS | RAIC | LTIS | LTIS |
|  | 860 | L | L | V | L | V | L | L |
|  | 879 | L | L | V | L | V | L | L |
|  | 881-883 | ASV | ASV | GSE | ASV | GSE | ASV | ASV |
|  | 895 | R | R | R | G | R | G | R |
|  | 900-903 | DLLF | DLLF | ALVV | DLLF | ALVV | DLLF | DLLF |
|  | 911-913 | LGT | LGT | RGG | LGT | RGG | LGT | LGT |
|  | 969 | A | A | A | A | A | G | A |
|  | 970 | A | A | T | A | A | A | A |
|  | 1192 | T | T | T | T | T | A | T |
|  | 1267 | N | N | S | N | S | S | N |
|  | 1276 | N | N | T | N | T | T | N |
|  | 1281 | N | N | T | N | T | T | N |
|  | 1294 | F | F | L | F | L | L | F |
|  | 1297-1298 | NS | NS | TT | NS | TT | TT | NS |
|  | 1304 | F | L | L | L | L | L | L |
|  | 1318 | V | L | L | L | L | L | L |
| ORF3 | 70 | D | － | D | D | － | － | D |
|  | 74 | I | V | I | I | I | I | I |
|  | 100-143 | / | / | △44aa | / | △44aa | / | / |
|  | 144-152 | IQLDE  YATI | IQLDE  YATI | STRRV  CYN* | IQLDE  YATI | STRRV  CYN* | IQLDE  YATI | IQLDE  YATI |
| E | 16-20 | LWLFV | LWLFV | LWLFV | ----- | LWLFV | ----- | ----- |
|  | 25 | L | L | L | P | L | P | P |
| M | 10 | E | E | E | E | A | E | E |
|  | 23 | N | N | T | N | N | N | N |
|  | 77 | V | F | V | V | V | V | V |
|  | 126 | T | I | I | T | I | T | T |
| N | 18 | L | L | L | L | F | L | L |
|  | 24 | V | G | V | V | V | V | V |
|  | 182 | Q | Q | R | Q | Q | Q | Q |
|  | 403 | C | Y | C | C | C | C | C |
|  | 441 | N | S | N | N | N | N | N |

*: termination codon; ---/△: amino acid (aa) deletion;

/: the same with the parent strain;
