# Supplementary figures and images for: Identification of two mutation sites in spike and envelope proteins mediating optimal cellular infection of porcine epidemic diarrhea virus from different pathways
Source: Vet Res. 2017 Aug 30;48:44. doi: 10.1186/s13567-017-0449-y (PMC5577753; doi:10.1186/s13567-017-0449-y)

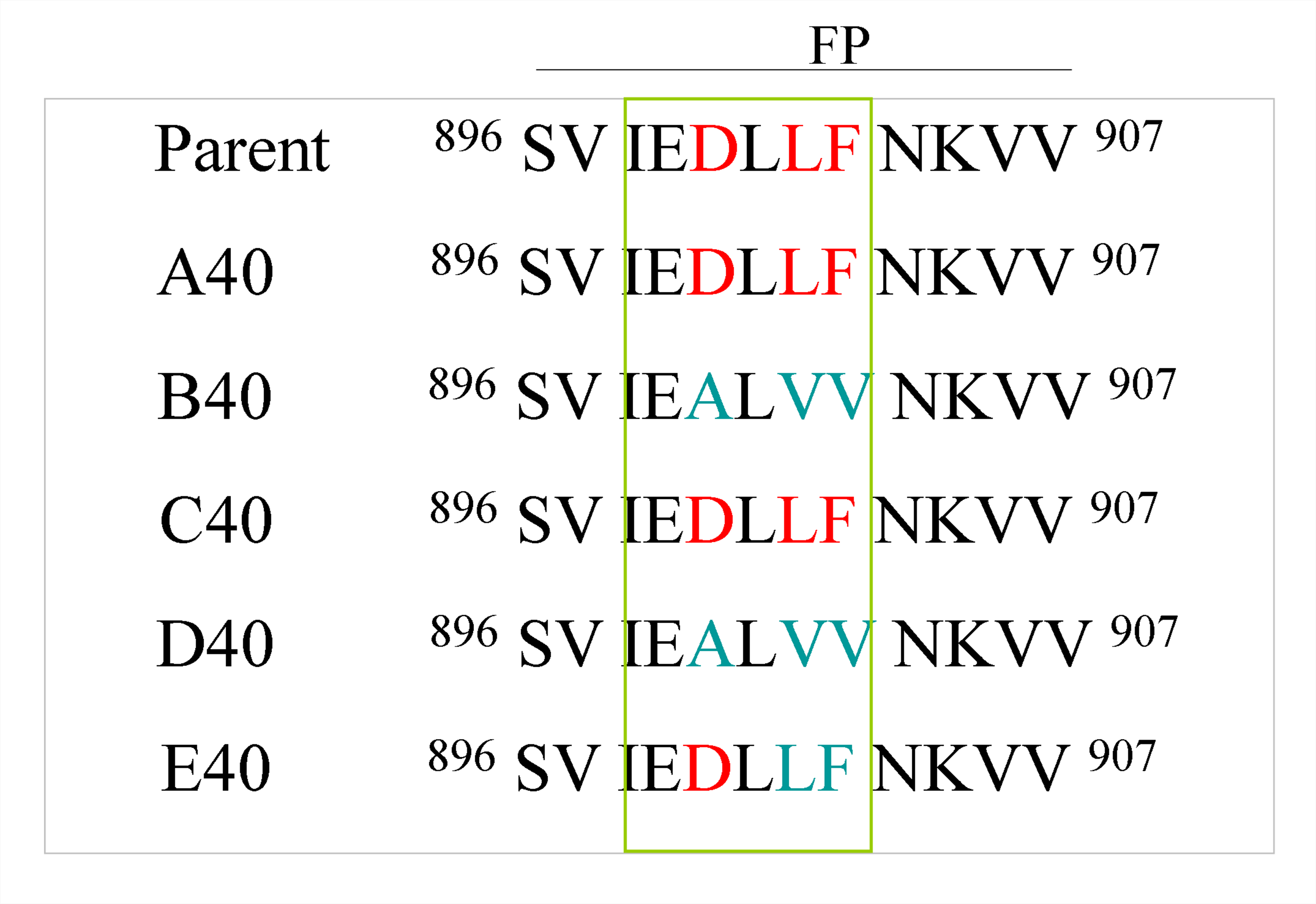

Supplement: Supplementary file 4 — Additional file 4. The alignment of the amino acid sequences 898 IEDLLF 903 in the fusion peptide (FP). Minimal divergence with occasional conservative substitutions in B40 and D40 strains were shown. The amino acids of I, Isoleucine; L, Leucine; F, Phenylalanine; V, Valine were hydrophobic amino acids. The E, Glutamic acid, and D, Aspartic acid were negative-charged amino acids. [file 13567_2017_449_MOESM4_ESM.tif]

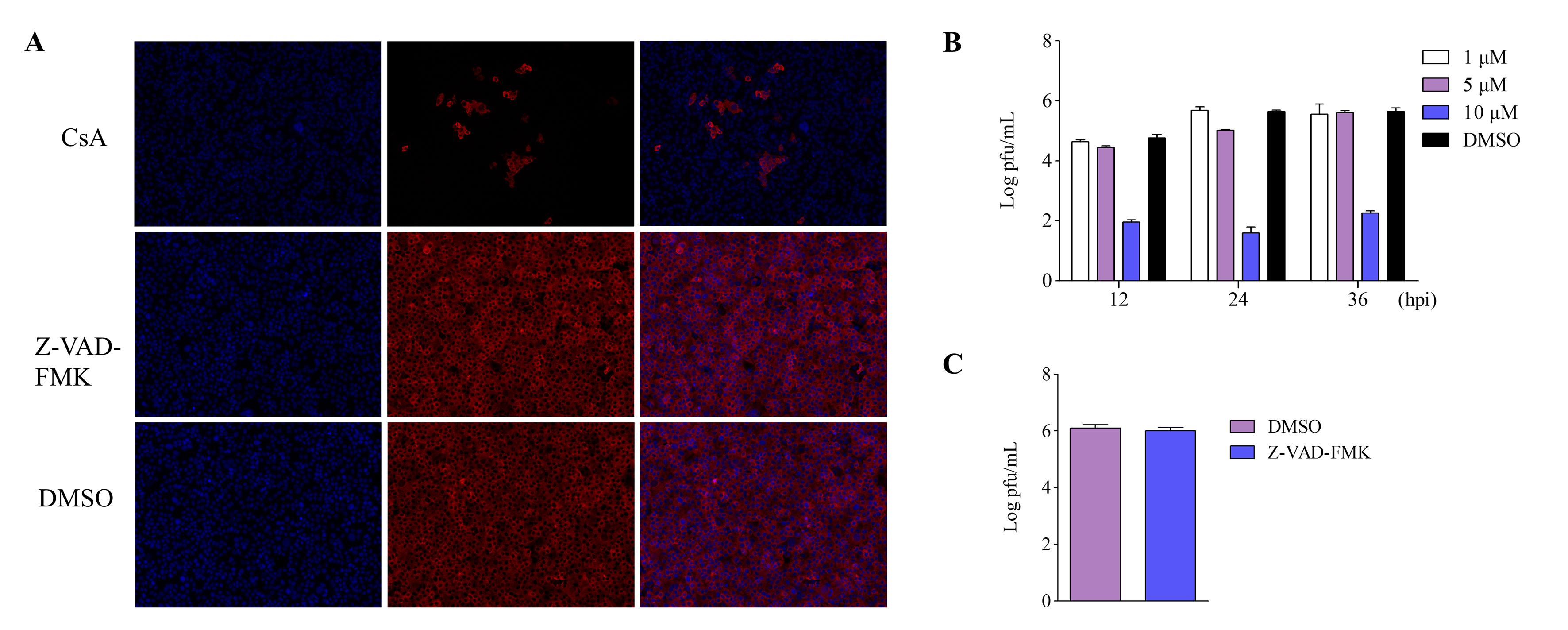

Supplement: Supplementary file 5 — Additional file 5. PEDV strain 85-7 induced caspase-independent apoptosis on Vero cells. (A) IFA of 85-7 parent strain in infected Vero cells with treatment of CsA (10 μM), V-ZAD-FMK (100 μM) and DMSO at 36 hpi. CsA treatment suppressed PEDV replication, while the V-ZAD-FMK had no significant effect on virus growth. Vero cells were pretreated with CsA, V-ZAD-FMK or DMSO for 1 h, and then infected with PEDV with the presence of CsA, V-ZAD-FMK or DMSO in the whole infected process. (B) Viral titers of the infected cells with CsA (1, 5, 10 μM) or DMSO treatment at 12, 24 and 36 hpi. Viral titers were determined as Log pfu/mL. Error bars indicate the average results of two independent experiments. (C) Viral titers of the infected cells with V-ZAD-FMK (100 μM) or DMSO treatment at 36 hpi. [file 13567_2017_449_MOESM5_ESM.tif]

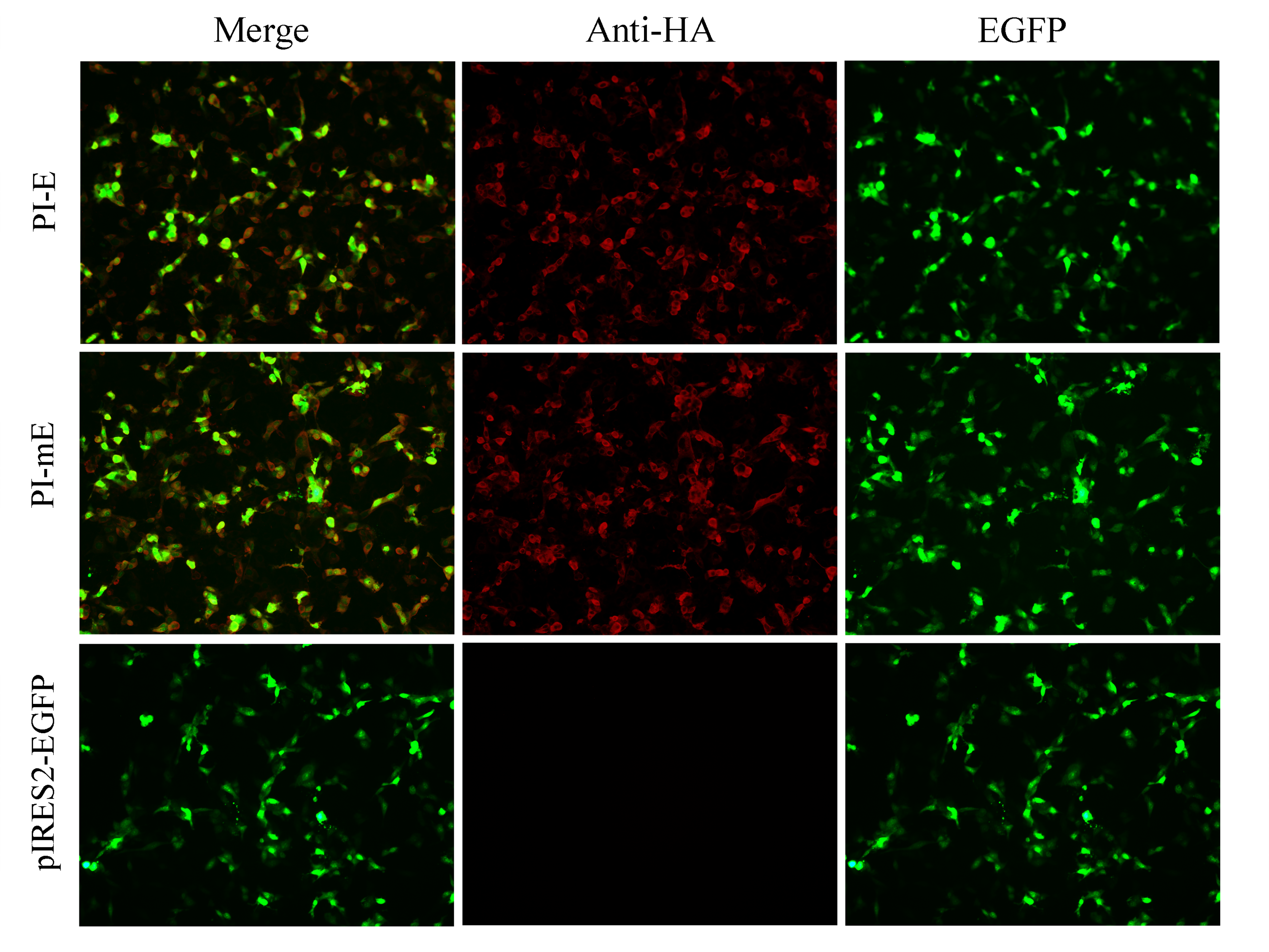

Supplement: Supplementary file 6 — Additional file 6. The dual-staining IFA assay to verify the overexpression of PEDV parent E protein and mutant E proteins in Vero cells. The recombinant plasmids (PI-E or PI-mE) were transfected into Vero cells for 24 h. The corresponding amount of empty plasmid (pIRES2-EGFP) was used as the mock control. The primary antibody was the anti-HA-tag antibody. The red staining (recombinant protein) was almost merged with the green staining (EGFP-tag protein). [file 13567_2017_449_MOESM6_ESM.tif]

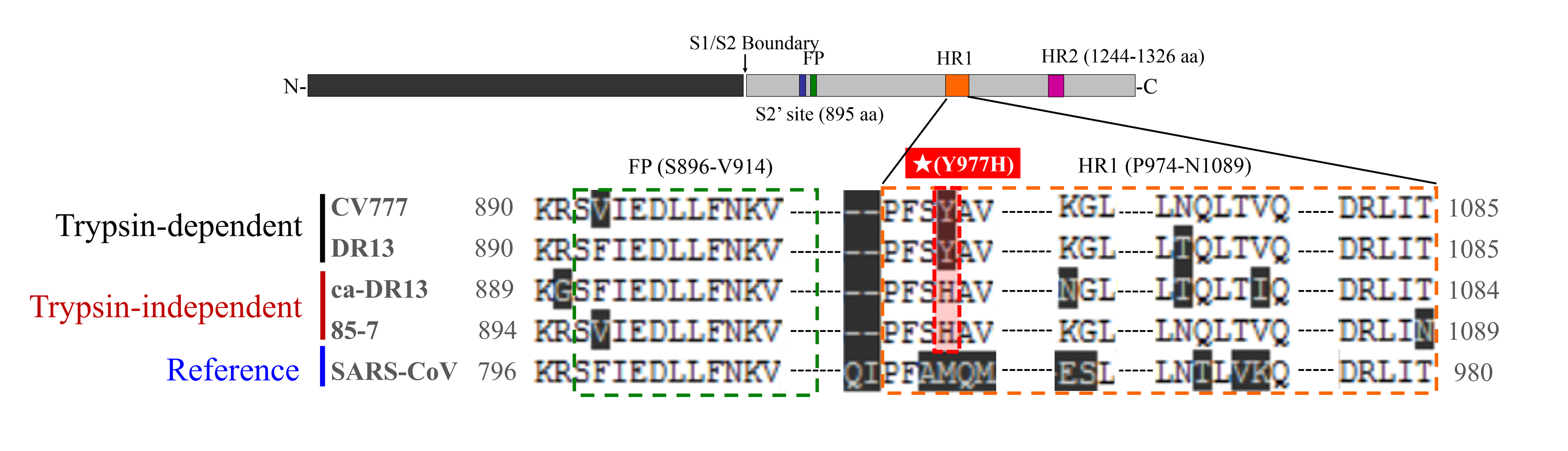

Supplement: Supplementary file 7 — Additional file 7. Multiple amino acid sequence alignment of the determinant region for PEDV trypsin-dependent entry. A schematic overview of the S2 domain is referred to as the SARS-CoV. FP, fusion peptide; HR1 and HR2, heptad repeat regions; S2′ site (R895), location of putative cleavage site within the S2 subunit; drawn to scale. Virus abbreviations (and GenBank accession numbers) were as follows: SARS-CoV (Tor2, NP_828851.1), DR13 (JQ023161.1), ca-DR13 (JQ023162.1), CV777 (AF353511.1). The Y977H was the only site that differed the trypsin-independent strains (ca-DR13 and 85-7 strain) from the trypsin-dependent strains (DR13 and CV777). [file 13567_2017_449_MOESM7_ESM.tif]
